# Reliability and Validity of the Migraine Disability Assessment Scale among Migraine and Tension Type Headache in Iranian Patients

**DOI:** 10.1155/2014/978064

**Published:** 2014-01-16

**Authors:** Alireza Zandifar, Fatemeh Asgari, Faraidoon Haghdoost, Samaneh Sadat Masjedi, Navid Manouchehri, Mahboobeh Banihashemi, Abbas Ghorbani, Mohammad Reza Najafi, Mohammad Saadatnia, Richard B. Lipton

**Affiliations:** ^1^Physiology Research Center, Department of Physiology, Isfahan University of Medical Sciences, Isfahan, Iran; ^2^Medical Student Research Center, Isfahan University of Medical Sciences, Isfahan, Iran; ^3^Department of Neurology and Isfahan Neurosciences Research Center, Isfahan University of Medical Sciences, Isfahan, Iran; ^4^Albert Einstein College of Medicine, Bronx, NY, USA; ^5^Montefiore Medical Center, Bronx, NY, USA

## Abstract

*Introduction*. MIDAS is a valid and reliable short questionnaire for assessment of headache related disability. Linguistic validation of Persian MIDAS and assessment of psychometric properties between tension type headache (TTH) and migraine were the aims of this study. *Methods*. Patients with migraine or TTH were included. At the first visit, we administered a headache symptom questionnaire, MIDAS, and SF-36. Patients filled out MIDAS in second and third visit within three and eight weeks after base line visit. Internal consistency (Cronbach **α**) and test-retest reproducibility (Spearman correlation coefficient) were used to assess reliability. Convergent validity and MIDAS capability to differentiate between chronic and episodic headaches (migraine and TTH) were also assessed. *Results*. The 267 participants had episodic migraine (EM-64%), chronic migraine (CM-13.5%), episodic TTH (ETTH-13.5%), and chronic TTH (CTTH-9). Internal consistency reliability was 0.8 for the entire sample, 0.72 for TTH, and 0.82 for migraine. Test-retest reliability for all questions between visit 1 and visit 2 varied from 0.54 to 0.71. Convergent validity was assessed using SF-36 as an external referent. Patients with episodic headaches (EM and ETTH) had significantly lower MIDAS scores than chronic headaches (CM and CTTH). *Conclusion*. Persian MIDAS is a valid and reliable questionnaire for migraine and TTH that can differentiate between episodic headache and chronic headache.

## 1. Introduction

Migraine and tension type headaches (TTH) are of the most common primary headache disorders that affect about 15% and 40% in general population, respectively, with a higher prevalence between the ages of 25 and 55. Migraine is characterized by frequent attacks of moderate to severe intensity, associated with autonomic symptoms [1, 2]. It also limits daily activities, impairs professional and educational performance and affects activities in family and society [3–5]. Frequency, duration, and severity of headaches and associated symptoms differ among patients and from one attack to another [6–8]. Different questionnaires are available to quantify different aspects of migraine, such as migraine-specific quality of life questionnaire (MSQ) that evaluates quality of life in migraine patients [9, 10], migraine severity (MIGSEV) scale that accesses severity of pain in different attacks [11], headache impact test (HIT-6) that measures impact of headache during one month [12], and migraine disability assessment scale (MIDAS) that measures disability related to migraine in a three-month period [13].

World Health Organization (WHO) defined disability adjusted life years (DALY), as an important indicator of disability. Migraine is one of the most disabling disorders in the world with a high score of DALY. Therefore, quantification of the disability in migraine patients is of great importance for both the patient and the physician in order to determine disease severity and to choose appropriate treatment [1, 14–17].

The migraine disability assessment (MIDAS) questionnaire has been developed by Lipton et al. [13] and can be used to assess all associated aspects with migraine and TTH disability in different domains of life. It has been extensively studied and its reliability and validity have been proved by standard methods in many countries [18, 19]. 

Regarding the lack of a valid and reliable version of MIDAS in Iranian population, the aim of this study was linguistic validation and assessment of psychometric properties of Persian version of MIDAS questionnaire. We also assessed and compared psychometric properties of MIDAS questionnaire in Iranian patients with migraine and TTH. MIDAS capability for discrimination between chronic and episodic headaches was also evaluated.

## 2. Methods and Materials

### 2.1. Patients and Settings

The subjects were diagnosed (migraine or TTH) and selected from four clinics in Isfahan, Iran, by four neurologists with at least 10-year experience in field of neurology that also had been participated in the pilot study. Patients participated in the study for an eight-week period. The patients were diagnosed based on International Headache Society criteria (IHS) as migraine or tension type headache [20]. All of the neurologists were checked by the corresponding author to diagnose based on IHS. According to the frequency of headache in a month, the migraine and TTH patients were classified as episodic (less than 15 headache episodes per month) and chronic (more than 15 headache episodes per month) [20]. Patients with medication overuse headache (MOH) also were selected based on IHS criteria for MOH [21].

Participants were asked to complete the questionnaire in the first day (visit 1), 3rd week (visit 2), and 8th week after the enrollment. The patients' condition was stable in all three visits and the stability was defined based on the neurologists' opinion as no need for any modification in drug type or dosage (all subjects were under treatment by at least one prophylactic and one analgesic drug). The patients that needed any changes in types or dosages of drugs were excluded from the rest of the study.

The sociodemographic and headache characteristics of all the subjects including age, sex, marital status, living place, education, frequency and duration of headaches, and aura were asked in the first visit (baseline). SF-36 questionnaire was also answered in the first visit.

Severity of headaches in all three visits was described by patients on a scale from zero to ten; zero described no existence of pain and ten showed the worst pain that they had ever experienced.

### 2.2. Assessment

MIDAS questionnaire is a short, self-administered questionnaire designed to quantify headache-related disability over a 3-month period. The MIDAS score is based on five disability questions in three dimensions: questions one and two assess the number of missed or significant limitations to activity (defined as at least 50% reduced productivity) days due to headache in school or paid work activities (school/job dimension); questions three and four assess the number of missed or significant limitations to activity (defined as at least 50% reduced productivity) days due to headache in housework activities (housework dimension); question five assesses missed days due to headache in family, social, or leisure activities (social dimension). The MIDAS score is the sum of responses to questions one through five. Two supplemental questions (A and B) provide the physician with additional clinical information about headache frequency and the average pain intensity (scale from zero to 10) of headaches over the previous three months [22].

SF-36 is a generic health survey that consists of two components of mental and physical wellbeing. Physical component is assessed through four dimensions: physical functioning (10 questions), role-physical (4 questions), bodily pain (2 questions), and general health (5 questions). Mental component is assessed through the other four dimensions: vitality (4 questions), social functioning (2 questions), role-emotional (3 questions), and mental health (5 questions). Each dimension and each major field of the questionnaire score from zero to 100. Higher scores stand for better quality of life. SF-36 questionnaire has been already translated into more than a hundred different languages. The validity and reliability of Persian version of SF-36 have been proven by Montazeri et al. in 2005 [23].

### 2.3. Linguistic Validation

Permissions were acquired from the corresponding author of original MIDAS article. A standard multistep forward-backward method was used to translate MIDAS to Persian. During the translation process several sessions were held with the presence of translators, two neurologists, and an expert in linguistics. Feedbacks were sent to the developers of original MIDAS questionnaire and their comments were used to edit the Persian version of MIDAS. For cognitive debriefing and clinicians' review, 20 migraine patients and five neurologists were enrolled in a pilot study using the latest version of Persian translation of MIDAS. The final Persian MIDAS questionnaire was produced using the data provided by both the pilot study and comments from Dr. R. B. Lipton, one of the original developers of MIDAS questionnaire [22].

### 2.4. Psychometric Analysis

#### 2.4.1. Reliability

Cronbach *α* and Spearman correlation coefficient were used to assess internal consistency and test-retest reliability of MIDAS questionnaire. Within three and eight weeks after the first visit (baseline), patients filled out MIDAS questionnaire and Cronbach *α* was calculated for first, second, and third visits separately. The results from the first two visits were used to calculate Spearman correlation between all individual questions. Results were also used to measure Spearman correlation of total scores between first and second, second and third, and also first and third visits. Both Spearman correlation and Cronbach *α* were calculated among all patients and two groups of TTH and migraine separately. To check for stable status of headaches, we compared mean score of each question between first and second visits.

#### 2.4.2. Validity

Convergent validity of MIDAS was analyzed through the assessment of correlation between MIDAS and SF-36 scores. Correlations were assessed between MIDAS (MIDAS total score, three different dimensions of MIDAS, and each individual question) and SF-36 questionnaire (eight dimensions and two different components). Also to analyze convergent validity, we assessed correlation between MIDAS scores and the number of headache days per month (HDPM) and MIDAS B question as numeric rating scale (NRS).

Convergent validity was only assessed in the baseline data, in all patients, and in two groups of TTH and migraine separately. We expected that higher scores in MIDAS questionnaire accompany lower scores in SF-36 and subsequently higher numbers of HDPM and NRS.

Item discriminant validity of MIDAS was assessed in a two-step process, analyzing the correlations between each individual question and its related dimension and also each individual question and total MIDAS score.

#### 2.4.3. Discrimination between Chronic and Episodic Migraine and Tension by MIDAS

Total scores and scores from each dimension of MIDAS were compared between episodic tension type headache (ETTH), chronic tension type headache (CTTH), chronic migraine (CM), and episodic migraine (EM). We assumed that no significant difference would be detected between ETTH and EM and also between CTTH and CM, but significant differences between chronic headaches (CM and CTTH) and EM also ETTH patients were anticipated.

#### 2.4.4. Ethics

The research protocol was approved by the Ethical Committee of Isfahan University of Medical Sciences in Iran.

#### 2.4.5. Data Analysis

Cronbach *α* was used to measure internal consistency. *α* ≥ 0.7 and ≥0.8 was considered the acceptable and excellent internal consistency, respectively.

Spearman correlation was used in test-retest reliability, convergent, divergence, item discriminant validity, and responsiveness correlation calculations. As MIDAS is not normally distributed, the Spearman correlation coefficient (*r*) was reported. We assumed 0.2 < *r* < 0.4 as a low to modest correlation, 0.4 < *r* < 0.5 as moderate correlation, and *r* > 0.5 as highly correlation. The Student's *t*-test was used to compare the mean scores between two groups. ANOVA was used to compare the mean scores between groups.

## 3. Results

### 3.1. Patients and Clinical Characteristics

From four clinics in Isfahan a total of 274 patients (26.3% males, mean age = 31.03 ± 9.41 years) were enrolled in the study and 267, 205 and 59 patients had participated in visit 1, visit 2, and visit 3, respectively. Patients and headache characteristics are shown in Tables 1 and 2, respectively. All patients were classified into four groups: EM, CM, ETTH, and CTTH groups including 64% (*n* = 171), 13.5% (*n* = 36), 13.5% (*n* = 36), and 9% (*n* = 24) of the participants, respectively. In chronic patients 66.6% of them suffered from MOH.

### 3.2. Reliability

The internal consistency of the questionnaire in first, second, and third visits was assessed using Cronbach *α* and was 0.80, 0.77, and 0.65, respectively. For TTH and migraine headache patients in first visit, the internal consistency analysis revealed Cronbach *α* of 0.72 and 0.82, respectively.

Test-retest reliability was tested for all of the patients with stable condition at visit 2 comparing with visit 1. The mean total MIDAS score in first and second visits were 32.56 ± 2.58 (median = 20 days) and 30.82 ± 2.54 (median = 20 days), respectively, (*r* = 0.71, *P* value <0.001) (Table 3).Correlation of total MIDAS score between visits 1 and 3 was 0.65 (*P* < 0.001) and between visit 2 and 3 was 0.77 (*P* < 0.001). Test-retest reliability analysis of MIDAS for TTH and migraine patients is also reported in Table 3.

### 3.3. Validity

Correlations of each question with total MIDAS score and also with its dimension are shown in Table 4. All questions were significantly correlated with the total MIDAS score and the related dimension (*P* < 0.001).

### 3.4. Convergent Validity

Total MIDAS score in the first visit was negatively correlated with SF-36 mental and physical scores in total patients (*r* = −0.41 and −0.36, resp., *P* < 0.001), migraine patients (*r* = −0.38 and −0.38, resp., *P* < 0.001), and TTH patients (*r* = −0.50 and −0.26, resp., *P* < 0.01).  Table 5 is reporting the correlation of all MIDAS questions and dimensions with SF-36 mental and physical scores and also eight dimensions of it in total patients.

Total MIDAS score in the first visit was positively correlated with the headache days per month (HDPM) of total, migraine and tension patients (*r* = 0.59, 0.61, and 0.56, resp., *P* < 0.001) (Table 6). Correlation of total MIDAS score with NRS score in the total, migraine, and TTH patients was also analyzed (*r* = 0.36, 0.29, and 0.60, resp., *P* < 0.001). Table 6 also shows the correlation between total MIDAS score with NRS and HDPM for all three dimensions.

### 3.5. Discrimination between Episodic and Chronic Headaches by MIDAS

Comparison of EM, ETTH, CM, and CTTH according to total MIDAS score and the three dimensions showed that there are no significant differences within chronic headache patients (CM and CTTH). Also we found no significant differences within episodic headache patients (EM and ETTH). But there are statistically significant differences between chronic and episodic headaches patients (Table 7).

### 3.6. Discrimination between Chronic Headaches with MOH and without MOH

Comparison of total MIDAS scores and the three dimensions between patients with MOH and without MOH are reported in Table 8. Total MIDAS score and the three dimensions were not significantly different between MOH and without MOH group.

## 4. Discussion 

The migraine disability assessment (MIDAS) questionnaire is a valid and reliable instrument to evaluate different aspects of migraine and TTH caused disability. Our results were consistent with the prior studies on MIDAS validation.

Based on our results, reduced or missed days of housework (housework dimension) had higher scores, while school/job reduced or missed activity (school/job dimension) had lower scores. Our study population was selected consecutively and the majority of the participants were female (73.7%). This explains the higher scores in housework activity impairment, since most of the Iranian women are housewives. Results from other studies in countries similar to Iranian population, such as Turkish study [14], match our findings, while studies in countries with western life style report a different set of results [1, 16]. Also we found that reduced housework and school/job activity are higher than missed days of housework or school/job activity, which is consistent with the most of the studies [1, 22].

### 4.1. Reliability

We evaluated and confirmed MIDAS internal consistency, using Cronbach *α*  (*α* = 0.8). Our results match the findings reported by prior studies [16]. We also found satisfactory internal consistencies in both TTH and migraine patients.

We compared mean of total MIDAS scores between first and second visits in all patients to evaluate test-retest reliability of MIDAS. Excellent correlations were found between total MIDAS scores of first and second visits in all patients (*r* > 0.7). Proper correlations (*r* > 0.5) were found between individual questions of MIDAS. We found a higher level of test-retest reliability among migraine patients rather than TTH patients, which points to a more stable condition among migraine patients because TTH per se is affected by multiple factors such as psychological items [19, 24].

### 4.2. Validity

As anticipated there was a significant (*r* > 0.4) correlation between each question and total MIDAS score; however, the correlation between each individual question and its related dimension was much stronger (*r* > 0.7).

Higher MIDAS scores were accompanied by lower scores in SF-36 as a proof to convergent validity. Both physical and mental components of SF-36 had negative correlations with MIDAS total scores (*r* > 0.2, *P* < 0.001). Modest correlations can be justified if we consider that SF-36 is a general tool, while MIDAS is specially designed for evaluation of headache disability. In our study mental component of SF-36 had a more obvious relation with both migraine and TTH MIDAS scores in comparison to physical component of SF-36. These results support prior research indicating that the burden of migraine on mental health is more prominent than physical health [25, 26]. However, in this study the association was higher in TTH than migraine. There was a slightly positive correlation between total MIDAS scores and HDPM when it had a low positive correlation with NRS; however, HDPM had a higher level of correlation. This may be due to the nature of HDPM and MIDAS as they measure frequency of headaches and headache related disability, while NRS assesses the severity of headaches.

### 4.3. Discrimination between Types of Headache

We assessed the capability of MIDAS to discriminate different types of headache. As we anticipated, total MIDAS scores were not significantly different between EM and ETTH and also CM and CTTH, but there were significant differences between different types of episodic and chronic headaches. Data of other researches such as Bigal et al. studies also were similar to our results and showed that CM is more disabling than episodic migraine [27, 28].

The total MIDAS score was not significantly different between patients with MOH and without MOH. In contrast to our results, data from another study that compared productivity loss in patients with and without analgesic overuse showed that medication overuse was associated with greater disability and productivity loss [29]. Probably the sample size of MOH patients in our study was not sufficient to show the difference between the groups, so further studies with higher sample size are needed to evaluate the effect of MOH on disability of migraine patients.

We categorized our patients as EM, CM, ETTH, or CTTH based on the diagnosis of a neurologist, which decreases the probability of misclassification. Then again our participants were collected from specialty clinics. This could cause a selection bias as these patients might have more severe cases of headache; however, this helps to reassure the beneficial use of MIDAS as a valid and reliable tool in health care delivery in clinical practice [19]. Another point of strength in our study was that there were no differences in mean scores of each question and in total MIDAS score between first and second visits that show the stability of the headache in our participants.

In conclusion, we produced the first Persian version of MIDAS questionnaire through a standard method of linguistic validation and proved its validity and reliability to be used for evaluation of migraine and TTH patients. We also found that MIDAS can potentially distinguish episodic and chronic headaches. However, more population based studies with enough sample size are needed to assess the capability of MIDAS to differentiate between different types of headaches.

## Figures and Tables

**Table 1:** Demographic characteristics of the patients.

	*N*	%
Sex		
Male	72	26.3
Female	202	73.7
Marital status		
Single	78	29.5
Married	186	70.5
Living place		
Urban	163	62.7
Rural	97	37.3
Education		
Primary school	10	3.8
Guidance school	43	16
Diploma	136	50.5
Bachelor and above	80	29.7

**Table 2:** Clinical characteristics of the patients.

	*N*	%
Frequency of headache		
Every day	11	4.2
More than once a week	141	55.2
Once a week	25	9.7
Less than once a week	79	30.9
Duration of headache		
≤3 hours	229	92.4
3–24 hours	19	7.6
>24 hours	0	0
Aura (in migraine patients)		
Yes	49	26.6
No	135	73.4

**Table 3:** Mean MIDAS score by question and overall with test-retest reliability estimates by headache type.

	Visit 1 all patients	Visit 2 all patients	*r*	Visit 1 migraine patients (*n* = 207)	Visit 2 migraine patients (*n* = 156)	*r*	Visit 1 TTH patients (*n* = 60)	Visit 2 TTH patients (*n* = 49)	*r*
Q1	2.99 ± 0.48	3.33 ± 0.54	0.62**	3.05 ± 0.48	3.86 ± 0.67	0.63**	2.50 ± 0.87	1.43 ± 0.63	0.58**
Q2	6.42 ± 0.81	5.79 ± 0.67	0.64**	6.53 ± 0.83	5.91 ± 0.78	0.68**	6.51 ± 1.79	5.32 ± 1.26	0.52**
Q3	6.86 ± 0.71	6.72 ± 0.71	0.59**	7.80 ± 0.79	6.88 ± 0.81	0.62**	6.38 ± 1.52	6.13 ± 1.56	0.47**
Q4	9.63 ± 0.95	8.40 ± 0.77	0.61**	9.33 ± 0.90	8.12 ± 0.81	0.52**	14.45 ± 2.85	9.5 ± 2.03	0.85**
Q5	7.32 ± 0.82	7.06 ± 0.78	0.54**	8.38 ± 0.99	6.74 ± 0.81	0.51**	7.16 ± 1.60	8.32 ± 2.10	0.58**
Total MIDAS score	32.56 ± 2.58	30.82 ± 2.54	0.71**	35.30 ± 3.15	31.19 ± 2.84	0.68**	36.27 ± 5.98	29.40 ± 5.67	0.85**

Q: question; TTH: tension type headache; M: migraine; data are given as Spearman correlation coefficient; **P < 0.001; each value is mean ± SE.

**Table 4:** The correlation of each question of MIDAS questionnaire with total score and its dimension.

Dimension	Question	Total MIDAS score	The question dimension
School/job	Q1	0.36*	0.71*
	Q2	0.51*	0.94*
Housework	Q3	0.72*	0.79*
	Q4	0.78*	0.91*
Social	Q5	0.73*	—

Data are given as Spearman correlation coefficient; *P < 0.001.

**Table 5:** The correlation of SF-36 components and dimensions with MIDAS scores.

	Physical functioning (PH)	Role physical (RP)	Bodily pain (BP)	General health (GH)	Vitality (VT)	Social functioning (SF)	Role emotional (RE)	Mental health (MH)	PCS	MCS
Q.1	−0.09*	−0.18**	−0.017**	−0.11*	−0.05	−0.21**	−0.10*	−0.06	−0.16*	−0.08*
Q.2	−0.09	−0.26**	−0.27**	−0.05	−0.18**	−0.24**	−0.21**	−0.11**	−0.16**	−0.17**
School/job dimension	−0.09	−0.28**	0.29**	−0.08	−0.16**	−0.27**	−0.21**	−0.11*	−0.18**	−0.17**
Q.3	−0.21**	−0.32**	−0.31**	−0.26**	−0.24**	−0.38**	−0.30**	−0.25**	−0.27**	−0.30**
Q.4	−0.33**	−0.36**	−0.30**	−0.21**	−0.32**	−0.37**	−0.36**	−0.28**	−0.28**	−0.33**
Housework dimension	−0.33**	−0.39**	−0.34**	−0.27**	−0.36**	−0.45**	−0.39**	−0.33**	−0.31**	−0.38**
Q.5 (social dimension)	−0.30**	−0.34**	−0.38**	−0.37**	−0.31**	−0.47**	−0.30**	−0.31**	−0.33**	−0.34**
Total MIDAS	−0.33**	−0.43**	−0.46**	−0.32**	−0.36**	−0.52**	−0.39**	−0.37**	−0.36**	−0.41**

Q: question; data are given as Spearman correlation coefficient; *P < 0.01; **P < 0.001.

**Table 6:** The correlation of numeric rating scale (NRS) and headache days per month (HDPM) with MIDAS score.

	Total patients (*n* = 267)		M (*n* = 207)		TTH (*n* = 60)	
	NRS	HDPM	NRS	HDPM	NRS	HDPM
School/job dimension	0.17**	0.23**	0.11*	0.28**	0.32**	0.09
Housework dimension	0.31**	0.48**	0.26**	0.51**	0.47**	0.44**
Social dimension	0.28**	0.48**	0.22**	0.50**	0.46*	0.45**
Total MIDAS score	0.36**	0.59**	0.29**	0.61**	0.60**	0.56**

NRS: numeric rating scale; HDPM: headache days per month; TTH: tension type headache; M: migraine; data are given as spearman correlation coefficient; *P < 0.01; **P < 0.001; *n*: number.

**Table 7:** Comparison of mean score of CTTH, ETTH, CM, and EM according to the total MIDAS score and the three dimensions.

	CTTH	ETTH	CM	EM	*P* value	*P* value					
						Comparison of CTTH with			Comparison of ETTH with		Comparison of CM and EM
						ETTH	CM	EM	CM	EM	
School/job dimension	10.83 ± 3.41	6.67 ± 1.77	18.08 ± 4.53	7.37 ± 0.95	0.003	0.741	0.281	0.794	0.011	0.983	0.002
Housework dimension	31.43 ± 7.11	14.34 ± 2.82	34.80 ± 6.32	13.66 ± 1.29	0.000	0.027	0.945	0.027	0.001	0.999	0.000
Social dimension	16.20 ± 4.10	3.73 ± 1.05	17.94 ± 4.22	6.38 ± 0.76	0.000	0.005	0.964	0.007	0.000	0.740	0.000
Total MIDAS score	58.65 ± 11.25	23.36 ± 4.66	70.83 ± 12.68	27.97 ± 2.44	0.000	0.012	0.701	0.007	0.000	0.941	0.000

CTTH: chronic tension type headache; ETTH: episodic tension type headache; CM: chronic migraine; EM: episodic migraine; each value is mean ± SE.

**Table 8:** Comparison of the total MIDAS score and the three dimensions between patients with MOH and without MOH within chronic headache patients.

	Chronic headache (migraine and tension type)		*P* value
	With MOH (39)	Without MOH (21)	
School/job dimension	14.48 ± 3.61	16.61 ± 5.68	0.754
Housework dimension	30.89 ± 5.37	32.55 ± 8.39	0.864
Social dimension	18.35 ± 4.02	14.95 ± 4.32	0.593
Total MIDAS score	63.74 ± 10.33	64.55 ± 17.08	0.966

MOH: medication overuse headache; each value is mean ± SE.
